# Effects of long-term magnesium supplementation on endothelial function and cardiometabolic risk markers: A randomized controlled trial in overweight/obese adults

**DOI:** 10.1038/s41598-017-00205-9

**Published:** 2017-03-07

**Authors:** Peter J. Joris, Jogchum Plat, Stephan J. L. Bakker, Ronald P. Mensink

**Affiliations:** 1 Department of Human Biology, NUTRIM School of Nutrition and Translational Research in Metabolism, Maastricht University Medical Center, Maastricht, 6200 MD The Netherlands; 2 The Top Institute of Food and Nutrition (TIFN), Wageningen, 6709 PA The Netherlands; 3 Department of Internal Medicine, University of Groningen, University Medical Center Groningen, Groningen, 9713 GZ The Netherlands

## Abstract

Long-term magnesium supplementation improves arterial stiffness, a cardiovascular disease risk marker. Effects on endothelial function may be another mechanism whereby increased magnesium intakes affect cardiovascular risk. Therefore, a 24-week, randomized, double-blind, placebo-controlled trial was performed to examine effects of magnesium supplementation on endothelial function and cardiometabolic risk markers. Fifty-two overweight and obese subjects (30 men and 22 women, age 62 ± 6 years) were randomized to receive either three times daily magnesium (total dose: 350 mg) or placebo capsules. Endothelial function was assessed at the start and at the end of the study. Cardiometabolic risk markers were measured at baseline, after 12 weeks, and at week 24. Brachial artery flow-mediated vasodilation did not change following long-term magnesium supplementation (0.49 *pp*; 95% CI: −0.38 to 1.36 *pp*; *P* = 0.26). Changes in reactive hyperemia index, retinal microvascular caliber and plasma markers for microvascular endothelial function (sVCAM-1, sICAM-1 and sE-selectin) were also not different. In addition, no effects on serum lipids, plasma glucose, insulin sensitivity, and low-grade systemic inflammation were observed. In conclusion, a daily magnesium supplement of 350 mg for 24 weeks does not improve endothelial function and cardiometabolic risk markers in overweight and obese middle-aged and elderly adults.

## Introduction

Prospective cohort studies have not only found an inverse association between dietary magnesium intake and diabetes^1^, but also with cardiovascular disease (CVD) risk^2, 3^. However, the number of well-designed intervention trials to examine a potential causal role of magnesium intake in the prevention of CVD is very limited. Recently, we have reported that in overweight and obese adults magnesium supplementation for 24 weeks resulted in a clinically relevant reduction in arterial stiffness, suggesting a potential mechanism by which dietary magnesium affects cardiovascular health^4^. Effects on endothelial function and cardiometabolic risk markers, which were secondary outcomes of the study, were however not reported. Conventional cardiometabolic risk markers are known determinants of arterial stiffness^5^ and the vascular endothelium has also been suggested to play an important role in arterial stiffening^6^. Reported effects of magnesium intake on serum lipids, plasma glucose, insulin sensitivity and low-grade systemic inflammation are inconsistent^7–11^, while only a few well-controlled intervention studies have examined effects on endothelial function. These trials involved patients taking anti-hypertensive drugs or medication known to affect lipid or glucose metabolism^12, 13^, which may have masked effects of oral magnesium supplementation, and only used markers reflecting large artery (i.e. the brachial artery) endothelial function^11–13^.

Endothelial function can be assessed in different ways. Brachial artery flow-mediated vasodilation (FMD), an ultrasound measurement of a large peripheral muscular artery, is considered the current non-invasive gold standard technique^14^. The change in pulse wave amplitude in response to blood flow-induced increases in shear stress is another functional marker of endothelial function, defined as the reactive hyperemia index (RHI). RHI reflects small artery reactivity^15^, while microvascular endothelial function can be assessed by measuring plasma markers that are synthesized by activation of the endothelium^16^. As these markers also relate to CVD risk^17^, effects on endothelial function of an increased magnesium intake were also assessed in our 24-week, randomized, double-blind, placebo-controlled intervention trial. The study involved overweight and obese middle-aged and elderly adults, because they are expected to have an impaired endothelial function^18^ and cardiometabolic disturbances at the start of the trial^19^, allowing for improvement by the intervention.

## Results

### Study subjects and compliance

The flow of participants through the study is shown in Supplemental Figure 1. A total of 51 subjects (29 men and 22 women) completed the trial. Baseline characteristics of these participants have been described before^4^. In brief, subjects were on average 62 ± 6 years old and their average BMI was 29.6 ± 2.8 kg/m^2^. Serum magnesium concentrations tended to increase in the magnesium compared with the placebo group by 0.02 mmol/L (95% CI: 0.00 to 0.04 mmol/L; *P* = 0.09) after 24 weeks, while twenty-four hour urinary magnesium excretion increased significantly by 2.01 mmol (95% CI: 1.22 to 2.93 mmol; *P* < 0.001) (Table 1). All subjects from the magnesium group had increased 24-hour urinary magnesium excretion concentrations at the end of the trial. This indicates that compliance of the subjects was excellent, as also evidenced from capsule counts. In fact, based on returned capsules, compliance ranged between 86% and 102%, and was on average >98% for the two treatment groups. No serious adverse events were reported in study diaries. Also, none of the participants has recorded in their study diaries any use of laxatives, which may contain magnesium oxide.

**Table 1:** Magnesium concentrations and vascular function measurements at baseline, and after a 12-week and 24-week magnesium or placebo treatment in a randomized controlled trial (RCT) with overweight and obese middle-aged and elderly adults^1^.

	Magnesium Group			Placebo Group			Treatment Effect	
	Baseline^2^	12 weeks^2^	24 weeks^2^	Baseline^2^	12 weeks^2^	24 weeks^2^	Δ 12 weeks^3^	Δ 24 weeks^3^
Magnesium concentrations								
Serum Mg, mmol/L	0.84 ± 0.05	0.87 ± 0.05	0.86 ± 0.04	0.85 ± 0.05	0.86 ± 0.04	0.85 ± 0.05	0.01 (−0.01; 0.04)	0.02 (0.00; 0.04)^#^
Urinary Mg, mmol/24 h	4.67 ± 1.15	N/A	6.55 ± 1.15	4.32 ± 1.44	N/A	4.28 ± 2.17	N/A	2.01 (1.22; 2.93)^##^
Vascular function								
Brachial artery diameter, cm	0.38 ± 0.05	N/A	0.38 ± 0.05	0.38 ± 0.06	N/A	0.39 ± 0.07	N/A	−1.39 (−3.19, 0.41)
Brachial artery FMD, %	3.11 ± 2.68	N/A	3.23 ± 2.57	3.75 ± 2.90	N/A	3.17 ± 2.15	N/A	0.49 (−0.38, 1.36)
Reactive hyperemia index	2.41 ± 0.61	N/A	2.57 ± 0.63	2.64 ± 0.48	N/A	2.57 ± 0.49	N/A	0.05 (−0.27, 0.37)
CRAE, μm	126 ± 17	124 ± 14	124 ± 15	128 ± 19	127 ± 20	125 ± 21	−1 (−4, 2)	1 (−1, 4)
CRVE, μm	223 ± 17	223 ± 15	222 ± 16	226 ± 19	225 ± 19	223 ± 21	1 (−2, 4)	1 (−1, 4)
Retinal AVR	0.56 ± 0.06	0.56 ± 0.05	0.56 ± 0.06	0.56 ± 0.06	0.56 ± 0.06	0.56 ± 0.06	−0.01 (−0.02, 0.01)	0.00 (−0.01, 0.01)
Endothelial dysfunction								
sVCAM-1, ng/mL	746 ± 122	N/A	742 ± 127	725 ± 155	N/A	708 ± 99	N/A	21 (−23, 65)
sICAM-1, ng/mL	467 ± 75	N/A	479 ± 79	449 ± 72	N/A	425 ± 71	N/A	14 (−17, 45)
sE-selectin, ng/mL	9.6 ± 4.5	N/A	11.8 ± 4.4	9.3 ± 5.0	N/A	11.3 ± 5.7	N/A	0.3 (−1.7, 2.3)

^1^Magnesium group: *n* = 26; placebo group: *n* = 25. Mg: magnesium; FMD: flow-mediated vasodilation; CRAE: central retinal arteriolar equivalent; CRVE: central retinal venular equivalent; AVR: arteriolar-to-venular diameter ratio; sVCAM: soluble vascular cell adhesion molecule; sICAM: soluble intercellular adhesion molecule; sE-selectin: soluble endothelial selectin; N/A: not available. ^2^Values are means ± SDs. ^3^Values are mean changes (95% CI) obtained from a one-way ANCOVA with baseline value as covariate. Treatment effect: ^**#**^ *P* < 0.10, ^**##**^ *P* < 0.001.

### Vascular function markers

**Figure 1:**
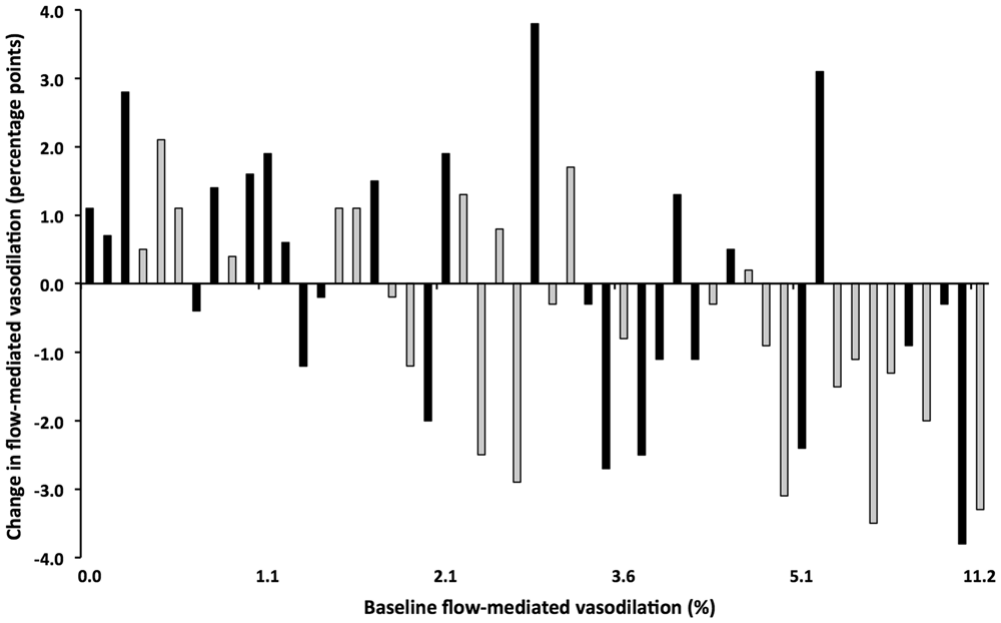
Individual changes in flow-mediated vasodilation. Effects of 24-week magnesium (black) and placebo supplementation (grey) on brachial artery flow-mediated vasodilation in overweight and obese middle-aged and elderly adults.

After 24 weeks of supplementation, changes in baseline brachial artery diameters were not statistically different between the two treatment groups (Table 1). Also, FMD did not change (Fig. 1). In addition, no effects of long-term magnesium supplementation on the RHI were found. Finally, the central retinal arteriolar equivalent (CRAE), central retinal venular equivalent (CRVE) and the retinal arteriolar-to-venular diameter ratio (AVR) did not change following long-term magnesium supplementation. Table 1 shows effects on plasma markers for microvascular endothelial function. No differences in soluble vascular cell adhesion molecule (sVCAM)-1, soluble intercellular adhesion molecule (sICAM)-1 and soluble endothelial selectin (sE-selectin) concentrations were observed.

### Cardiometabolic risk markers

Fasting total cholesterol, HDL-cholesterol, LDL-cholesterol, triacylglycerol, non-esterified fatty acid (NEFA), glucose, insulin and HOMA_IR_ did not differ after oral magnesium supplementation compared with the placebo treatment. The effects on plasma markers for low-grade systemic inflammation were also investigated. No effects were found on interleukin (IL)-6, IL-8, tumor necrosis factor (TNF)-α, C-reactive protein (CRP) and serum amyloid A (SAA) (Table 2). One man from the magnesium and four participants from the placebo group had CRP concentrations above 10 μg/mL on one (i.e. three CRP values at the start of the study and one at the end of the placebo treatment) or both occasions. Conclusions did not change when these participants were excluded form the statistical analyses (data not shown).

**Table 2:** Cardiometabolic risk measurements at baseline, and after a 12-week and 24-week magnesium or placebo treatment in a randomized controlled trial (RCT) with overweight and obese middle-aged and elderly adults^1^.

	Magnesium Group			Placebo Group			Treatment Effect	
	Baseline^2^	12 weeks^2^	24 weeks^2^	Baseline^2^	12 weeks^2^	24 weeks^2^	Δ 12 weeks^3^	Δ 24 weeks^3^
Cardiometabolic risk								
Total cholesterol, mmol/L	6.26 ± 0.96	6.34 ± 0.93	6.30 ± 0.97	5.70 ± 0.90	5.75 ± 0.87	5.66 ± 0.73	0.13 (−0.16, 0.41)	0.20 (−0.07, 0.48)
HDL-cholesterol, mmol/L	1.59 ± 0.49	1.60 ± 0.46	1.58 ± 0.48	1.39 ± 0.38	1.38 ± 0.32	1.36 ± 0.31	0.06 (−0.03, 0.14)	0.06 (−0.04, 0.16)
LDL-cholesterol, mmol/L	4.08 ± 0.79	4.10 ± 0.82	4.07 ± 0.89	3.59 ± 0.76	3.67 ± 0.71	3.61 ± 0.64	0.02 (−0.21, 0.25)	0.06 (−0.20, 0.32)
Triacylglycerol, mmol/L	1.30 ± 0.51	1.40 ± 0.56	1.41 ± 0.65	1.55 ± 0.65	1.55 ± 0.55	1.49 ± 0.58	0.04 (−0.16, 0.24)	0.11 (−0.12, 0.34)
NEFA, mmol/L^5^	430 ± 145	440 ± 144	436 ± 146	417 ± 126	382 ± 160	414 ± 145	50 (−19, 119)	15 (−55, 85)
Glucose, mmol/L^5^	5.91 ± 0.74	5.90 ± 0.80	5.97 ± 0.94	5.95 ± 0.63	5.88 ± 0.62	5.89 ± 0.66	0.05 (−0.15, 0.25)	0.12 (−0.09, 0.32)
Insulin, uU/mL^5^	12.1 ± 4.7	12.2 ± 4.7	12.7 ± 6.1	15.0 ± 5.3	14.1 ± 5.5	14.1 ± 6.1	0.6 (−1.0, 2.2)	1.5 (−0.6, 3.6)
HOMA_IR_^5^	3.19 ± 1.35	3.22 ± 1.37	3.41 ± 1.77	4.03 ± 1.69	3.74 ± 1.71	3.77 ± 1.86	0.23 (−0.22, 0.67)	0.48 (−0.12, 1.08)
Low-grade inflammation								
IL-6, pg/mL^4^	0.76 (0.62–0.97)	N/A	0.73 (0.65–0.97)	0.81 (0.64–1.02)	N/A	0.81 (0.65–1.12)	N/A	−0.07 vs. 0.00
IL-8, pg/mL^4^	3.75 (3.30–4.83)	N/A	4.55 (3.89–5.88)	4.28 (3.56–4.72)	N/A	4.60 (3.88–5.25)	N/A	0.56 vs. 0.36
TNF-α, pg/mL^4^	2.65 (2.25–2.97)	N/A	2.72 (2.40–3.16)	2.71 (2.18–2.96)	N/A	2.75 (2.29–2.95)	N/A	0.18 vs. −0.02
CRP, μg/mL^4,6^	1.59 (1.34–3.20)	N/A	1.77 (1.39–2.30)	1.72 (1.00–3.14)	N/A	1.65 (1.06–2.90)	N/A	−0.12 vs. −0.17
SAA, μg/mL^4,7^	3.84 (3.06–5.11)	N/A	4.45 (3.16–5.46)	3.79 (2.58–5.30)	N/A	2.94 (2.42–4.69)	N/A	0.40 vs. −0.15

^1^Magnesium group: *n* = 26; placebo group: *n* = 25. NEFA: non-esterified fatty acid; IL: interleukin; TNF: tumor necrosis factor; CRP: C-reactive protein; SAA: serum amyloid A; N/A: not available. ^2^Values are means ± SDs or medians (25–75^th^ percentile). ^3^For normally distributed variables, values are mean changes (95% CI) obtained from a one-way ANCOVA with baseline value as covariate. For non-normally distributed variables, variables are the median of the changes in respectively the magnesium and the placebo group. ^4^Markers for low-grade systemic inflammation were tested by a Wilcoxon rank-sum test for non-normal distributed data. ^5^Magnesium group: *n* = 25; placebo group: *n* = 25. ^6^Magnesium group: *n* = 25; placebo group: *n* = 21. ^7^Magnesium group: *n* = 24; placebo group: *n* = 22.

## Discussion

In this randomized controlled trial (RCT) involving overweight and obese middle-aged and elderly adults, we found no significant effect on FMD, RHI, retinal microvascular caliber and plasma markers for microvascular endothelial function after 24 weeks of daily supplementation with 350 mg magnesium. Also, cardiometabolic risk markers did not change following magnesium supplementation.

The lack of effect on FMD is in agreement with several^11, 12^, but not all^13^, earlier randomized, double-blind, placebo-controlled intervention trials examining effects of magnesium supplementation. In a 6-month study in 50 patients with stable coronary artery disease (CAD), a daily magnesium supplement of 365 mg increased FMD by 11.1 *pp* ^13^. The observed improvement by Shechter *et al*.^13^ is very pronounced and related with an estimated decrease of approximately 90% in the long-term risk to develop CVD^20^. In retrospect, we had a statistical power of 80% (*P* < 0.05) to detect a true change in FMD of at least 1.30 *pp*. Thus, our study was certainly adequately powered to detect such a huge effect. Differences in subject characteristics may have contributed to these inconsistent results. CAD may be associated with magnesium depletion^21^ and a magnesium-deficient state was indeed found in 36 of the 50 CAD patients^13^. Also, study participants had low intracellular magnesium concentrations, as assessed in sublingual epithelial cells. In our intervention trial and others involving also apparently healthy individuals^11^ or patients on hemodialysis^12^, participants had baseline serum magnesium concentrations within normal ranges. This suggests that a very specific population was studied by Shechter^13^.

Baseline diameters of the brachial artery did not change in our study. Consequently, changes in brachial diameters cannot explain our lack of effect on FMD. Also, no effects were found on the RHI. The RHI reflects small artery reactivity^15^, while FMD targets a large peripheral muscular artery^14^. To date, no other RCT has addressed the effect of magnesium supplementation on the RHI. Taken together, our study does not provide evidence that improvements in endothelial function have contributed to the beneficial effects on arterial stiffness associated with an increased magnesium intake for a 24-week experimental period, as reported previously^4^. As discussed^4^, long-term magnesium supplementation may primarily have an impact on the aorta and not on peripheral muscular arteries, possibly as a result of improvements in the structural characteristics of large elastic arterial walls. In fact, longer-term use of magnesium in patients receiving hemodialysis significantly decreased common carotid artery intima-media thickness^12, 22^, which reflects structural changes of the arterial wall^23^, without any apparent effects on FMD^12^.

Magnesium supplementation did also not affect retinal microvascular caliber. To the best of our knowledge, no other RCTs have assessed the effects of magnesium on microvascular diameters. Finally, markers for microvascular endothelial function were investigated in plasma, but no significant effects were observed for sVCAM-1, sICAM-1 and sE-selectin that are involved in the recruitment of leukocytes to the vascular wall^24^. In agreement, Chacko *et al*. observed that a daily magnesium supplement of 500 mg for four weeks did not change plasma concentrations of these markers in 14 otherwise healthy overweight individuals^10^.

Alternate possible mechanisms to explain the beneficial effects of oral magnesium supplementation on arterial stiffness in overweight and obese adults^4^ may relate to the postulated actions of magnesium on blood pressure and other cardiometabolic risk markers^25^. However, we have already reported that blood pressure did not change^4^, while serum lipids and lipoproteins, plasma glucose, insulin sensitivity and plasma markers for low-grade systemic inflammation were also comparable between the two treatment groups. A meta-analysis of nine RCTs involving patients with type II diabetes reported that magnesium supplementation (median daily dose: 360 mg) for 4 to 16 weeks increased HDL-cholesterol, but found no effects on total cholesterol, LDL-cholesterol and triacylglycerol concentrations^26^. In healthy subjects, however, there is no evidence that an increased dietary magnesium intake improves the serum lipid profile^9–11^. A recent meta-analysis and systematic review of 21 randomized trials summarized the effects of magnesium supplements (range: 300 to 600 mg/day) on glucose, insulin and HOMA_IR_ in both diabetic and non-diabetic individuals^27^. Study duration ranged from one to six months. In agreement, no effects were observed on fasting glucose and insulin. However, the HOMA_IR_ significantly decreased suggesting that insulin sensitivity improved following oral magnesium supplementation. These effects were more pronounced in individuals with hypomagnesemia as compared with normomagnesemic subjects, but did not depend on the presence of diabetes. Adults who completed the present trial had serum magnesium concentrations within normal range^28^, which may explain the lack of effect on the HOMA_IR_. Finally, no beneficial effects on pro-inflammatory cytokines were found. Similar conclusions were drawn in the very few RCTs that examined effects of magnesium supplementation on systemic inflammation in apparently healthy overweight adults^10, 29^.

Some limitations of the present trial warrant consideration. Our study was sufficiently powered to detect a change of 1.30 *pp* in FMD, while we estimated an effect of 0.49 *pp* (95% CI: −0.38 to 1.36 *pp*). Although the variability in our study was in line with findings of earlier studies^30^, we cannot exclude the possibility of false-negative finding. However, other markers of endothelial function did also not change and we therefore do no consider it very likely that oral magnesium supplementation improves endothelial function. Studies on underlying mechanisms are also needed, as the observed beneficial effects on large elastic arterial walls^4^ may relate to the postulated actions of magnesium on endothelial cells^31^. In fact, *in vitro* studies have shown that low extracellular magnesium induces the development of a pro-atherosclerotic phenotype of cultured endothelial cells^32^. Recent studies also suggest an important role of microRNAs on vascular function^33^ and the ability of endothelial cells to produce atherogenic catecholamines^34^. Broadening our knowledge in these novel fields will contribute to our understanding of mechanisms by which an increased oral magnesium intake beneficially affects cardiovascular health outcomes.

In conclusion, the present results indicate that a magnesium intervention for 24 weeks does not improve endothelial function and cardiometabolic risk markers in overweight and obese men and postmenopausal women. It is therefore unlikely that effects on endothelial function have contributed to the beneficial effects on arterial stiffness.

## Subjects and Methods

### Subjects and study design

Overweight and slightly obese men and postmenopausal women with a mean age of 62 ± 6 years participated in a randomized, double-bind, placebo-controlled, parallel study with a 24-week experimental period, as described previously^4^. In brief, study subjects were allocated to receive either three times daily magnesium (total dose: 350 mg; Magnesium Citrate Complex [Mg 16%]) or placebo capsules containing starch (Amylum Solani). A total daily dose of 350 mg is considered the tolerable upper intake level (UL) of supplemental magnesium for adults^35^. All capsules were kindly provided by Laboratorium Medisan B.V. (Heerenveen, The Netherlands). The capsules were prepared in one batch. Participants maintained their habitual diet, physical activity levels and consumption of alcohol throughout the total study period. Inclusion and exclusion criteria have been described before^4^. Briefly, all volunteers were apparently healthy and did not receive proton pump inhibitors, anti-hypertensive medication or drugs known to affect lipid or glucose metabolism. Fifty-two overweight and obese study subjects were included. They had a BMI between 25 and 35 kg/m^2^; serum creatinine concentrations <116 μmol/L for men and <101 μmol/L for women; and no indications for treatment with cholesterol-lowering medications^36^. All study participants gave written informed consent before the start of the trial. The study was approved by the Ethics Committee of Maastricht University Medical Center, and registered on 8 September 2014 at ClinicalTrials.gov as NCT02235805. The methods were performed in accordance with the described procedures in the approved study protocol.

### Blood sampling and analyses

Fasting blood samples were taken at the start of the study (days −3 and 0), at week 12 (day 84), and at the end of the study (days 165 and 168) from a forearm vein by venipuncture. On the days preceding blood sampling, participants were requested not to consume alcohol or to perform any strenuous physical exercise. On the morning of blood sampling, subjects arrived after an overnight fast (no food or drink after 08.00 PM, except for water) at the Metabolic Research Unit Maastricht (MRUM) research facilities by public transport or by car to the standardize measurements as much as possible. After blood sampling, NaF-containing vacutainer tubes (Becton, Dickinson and Company, Franklin Lanes, NY, USA) and EDTA-coated vacutainer tubes (Becton, Dickinson and Company) were immediately kept on ice and centrifuged within 30 minutes. To obtain plasma, plasma separator tubes were centrifuged at 1300× *g* for 15 minutes at 4 °C. Blood drawn in vacutainer serum tubes (Becton, Dickinson and Company) was first allowed to clot for at least 30 minutes at 21 °C. To obtain serum, serum separator tubes were centrifuged at 1300× *g* for 15 minutes at 21 °C. Following centrifugation, plasma and serum samples were immediately portioned into aliquots and stored at −80 °C until analysis at the end of the trial.

Fasting glucose (Horiba ABX SAS, Montpellier, France) and NEFA concentrations (NEFA kit; WAKO Chemicals GmbH, Neuss, Germany) were measured in NaF-plasma. Fasting serum samples were analyzed for total cholesterol (Cobas 8000; Roche Diagnostics Systems, Hoffmann-La Roche Ltd., Mannheim, Germany), HDL-cholesterol (Cobas 8000; Roche Diagnostics Systems), triacylglycerol (Cobas 8000; Roche Diagnostics Systems), and insulin concentrations (RIA; Millipore, Billerica, MA, USA). LDL-cholesterol was calculated using the Friedewald formula^37^. The degree of insulin resistance was estimated by calculating the HOMA_IR_^38^. Fasting EDTA-plasma samples were analyzed for markers for low-grade systemic inflammation (IL-6, IL-8, TNF-α, CRP, SAA) and markers for microvascular endothelial function (sVCAM-1, sICAM-1, sE-selectin) by using a multi-array detection system based on electro-chemiluminescence technology (SECTOR Imager 2400; Meso Scale Discovery, Rockville, MD, USA)^39^.

### Vascular function measurements

Measurements were performed at the start of the trial (day 0), at week 12 (day 84), and at the end of the study (day 168) in a quiet and darkened room. The room was temperature controlled at 22 °C. After blood sampling and an acclimatization period of 30 minutes in the supine position, measurements were performed.

FMD was assessed using ultrasound echography (SONOS 5500; Hewlett-Packard [Philips], Andover, MA, USA) and recording of echo images on DVD^40^. After a 5-minute reference period, the pneumatic cuff placed around the participant's forearm was inflated to 250 mmHg for 5 minutes, causing distal hypoxia. Upon cuff-release reactive hyperemia ensued. The echo images were analyzed offline using a custom-written Matlab program (MyFMD V14.07; Prof. A.P. Hoeks, Department of Biomedical Engineering, Maastricht University Medical Center, Maastricht, The Netherlands). The FMD response was quantified as the maximal percentage change in post occlusion arterial diameter relative to baseline diameter. During FMD measurements, the Endo-PAT 2000 (Itamar Medical Ltd, Caesarea, Israel) was used to measure changes in pulse wave amplitude in response to reactive hyperemia. In brief, a pneumatic probe was placed on the index finger of both hands to record the peripheral arterial tone, according to instructions of the manufacturer. The RHI was quantified as the post-to-pre occlusion peripheral arterial tone signal ratio in the occluded hand, normalized to values in the control hand and then further corrected for baseline vascular tone^41^. Finally, retinal vascular images were obtained to assess microvascular diameters in the eye. During this test, study participants were seated with their head resting on a chinrest, looking directly into the non-mydriatic retinal camera (Topcon TRC-NW-300; Topcon Co., Tokyo, Japan). The camera focused on the optic disc and photographed the retina. Images were digitized and analyzed to calculate the CRAE, CRVE, and retinal AVR with appropriate software (Generalized Dual-Bootstrap Iterative Closest Point [GDBP-ICP])^42^. In brief, the software automatically aligns the retinal images based on detected vascular centerlines by iteratively transforming the algorithm. At least two arteriolar and two venular retinal segments were measured and summarized by using the Parr-Hubbard formulas^43^. These segments had to be the same segments at each time point for an individual.

### Statistical analyses

Study results are presented as means ± SDs, unless otherwise indicated. A per protocol analysis was performed. Differences in baseline values between the magnesium and placebo group were tested using an unpaired Student's *t* test. A one-way ANCOVA, using the baseline measurements of the outcome variables as covariates, was conducted to investigate differences in responses between magnesium and placebo treatments. When residuals were not normally distributed as assessed with the Kolmogorov-Smirnov test, a Wilcoxon rank-sum was used. Changes were then calculated for each individual as the difference between the values at the end of the trial and at the start of the trial. A *P* *<* 0.05 was considered statistically significant. The study was powered on carotid-to-femoral pulse wave velocity, which was the primary outcome, but a retrospective power analysis showed that with 51 participants we had 80% power to detect a true change in FMD of at least 1.30 *pp*. For this power calculation, an alpha of 0.05 and observed within-subject variability in FMD of 1.64 *pp* were used. Analyses were performed using SPSS 23.0 (SPSS Incorporated, Chicago, IL, USA).

## Electronic supplementary material

Supplementary Information
