# Supplementary material for: Effects of long-term magnesium supplementation on endothelial function and cardiometabolic risk markers: A randomized controlled trial in overweight/obese adults
Source: Sci Rep. 2017 Mar 7;7:106. doi: 10.1038/s41598-017-00205-9 (PMC5428005; doi:10.1038/s41598-017-00205-9)
Supplement: Supplementary file 1 — Supplementary Information [file 41598_2017_205_MOESM1_ESM.doc]

**SUPPLEMENTARY INFORMATION**

Effects of long-term magnesium supplementation on endothelial function and cardiometabolic risk markers: A randomized controlled trial in overweight/obese adults

Peter J. Joris **^1,3,*^**, Jogchum Plat **^1^**, Stephan J.L. Bakker **^2^** and Ronald P. Mensink **^1,3^**

**^1^** Department of Human Biology, NUTRIM School of Nutrition and Translational Research in Metabolism, Maastricht University Medical Center, Maastricht 6200 MD, The Netherlands (P.J.J., J.P., R.P.M.)

**^2^** Department of Internal Medicine, University of Groningen, University Medical Center Groningen, Groningen 9713 GZ, The Netherlands (S.J.L.B.)

**^3^** The Top Institute of Food and Nutrition (TIFN), Wageningen 6709 PA, The Netherlands (P.J.J., S.J.L.B., and R.P.M.)

***** Corresponding author. Department of Human Biology, NUTRIM School of Nutrition and Translational Research in Metabolism, Maastricht University Medical Center, PO Box 616, 6200 MD Maastricht, The Netherlands. Tel.: +31-43-388-1311. E-mail address: p.joris@maastrichtuniversity.nl.


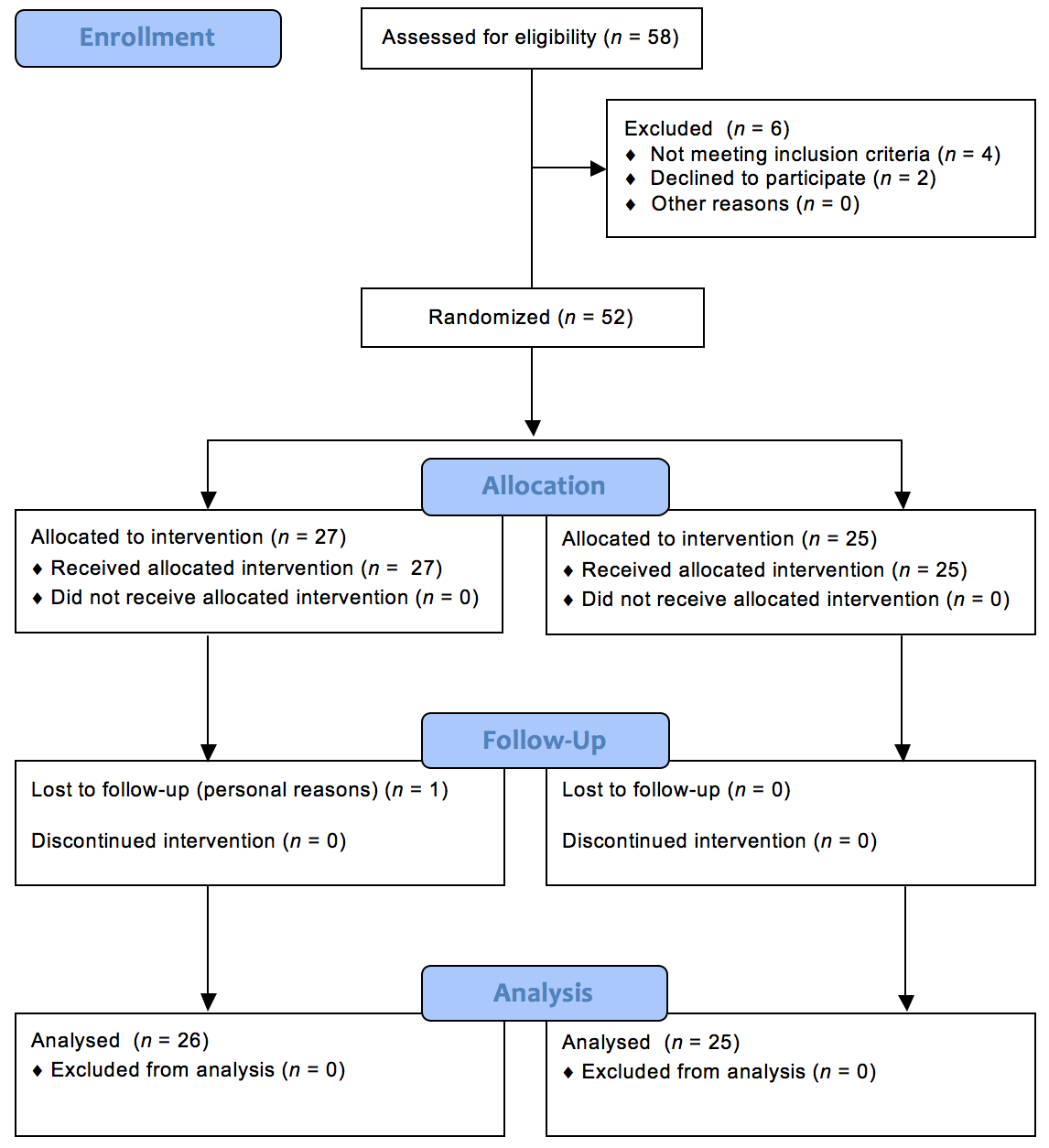


**SUPPLEMENTAL FIGURE 1**

Consort flow diagram [1]. Diagram of the progress through the phases of the present randomized parallel trial with two treatment groups.

**REFERENCES**

1. Schulz, K.F., Altman, D.G., Moher, D. & for the CONSORT Group. CONSORT 2010 Statement: updated guidelines for reporting parallel group randomised trials. *Trials* **11**, 32 (2010).
